# Phase transition of amorphous cobalt hydroxide to crystalline cobalt oxides by electron-beam irradiation

**DOI:** 10.1186/s42649-025-00107-5

**Published:** 2025-03-07

**Authors:** Minjeong Lee, Hye Seong Jang, Gayoung Yoon, Gyeong Hee Ryu

**Affiliations:** 1 Department of Materials Engineering and Convergence Technology, Gyeongsang National University, Jinju, 52828 Republic of Korea; 2 School of Materials Science and Engineering, Gyeongsang National University, Jinju, 52828 Korea

**Keywords:** Cobalt hydroxides, Cobalt oxides, Nanosheet, Electron beam

## Abstract

Transmission electron microscopy (TEM), which can analyze the shape and crystallinity of materials as well as the chemical bonding of ions and the states of elements, operates at different accelerating voltages depending on the type of specimen analyzed and the analysis area. Electron-beam irradiation can be used to induce structural transitions and crystallization of materials. Therefore, studies on phase transition using electron beams have been frequently conducted. Cobalt oxides, including cobalt hydroxides, have various phases and crystal structures, depending on their stoichiometric compositions. Specific synthesis methods can be used to synthesize these at low dimensions, in addition to large nanosheet structures. In this study, the crystallization and phase transition of amorphous cobalt hydroxide nanosheets induced by continuous electron-beam irradiation were analyzed using high-resolution TEM (HR-TEM). The synthesized cobalt hydroxide nanosheets were partially converted into cobalt oxides, and the transferred area expanded as the irradiation time increased. Under 300 kV of accelerating voltage, the transition to cubic cobalt oxides was dominant, suggesting a frequent transitional behavior of amorphous metal hydroxides upon electron-beam irradiation.

## Introduction

Cobalt oxides, including cobalt hydroxides and cobalt oxyhydroxides (Yang et al. 2010; Geng et al. 2008), and their derivative compounds have received considerable research interest owing to their exceptional chemical and physical properties. They are promising materials with wide applications in batteries (Do et al.^2005^; Yue et al. 2018), gas sensing (Frost et al. 2008; Yan et al. 2019), catalysts (Lai et al. 2008; Liu et al. 2014; Nam et al. 2017), among other applications (Liu et al. 2006; Zhang et al. 2008). Among them, cobalt hydroxide nanosheets exhibit excellent activity and durability when the phase is amorphous rather than crystalline (Liu et al. 2014; Koza et al. 2013). Moreover, that have good electrocatalytic properties (Gao et al. 2016; Babar et al. 2018). Among the various cobalt oxides, only Co_3_O_4_ and CoO (Ha et al. 2013) are stable and thus used in many processes. Co_3_O_4_ (Yoon et al. 2022; Chen et al. 2021; Deori et al. 2013) crystallizes into a cubic spinel structure. Cobalt monoxide (CoO), which has two phases, cubic and hexagonal (Alaria et al. 2008), is the simplest structure that crystallizes in a rock salt structure, is antiferromagnetic (Dai et al. 2013), and electrically insulating (Miranda-López et al. 2020) in bulk form at room temperature.

Cobalt oxide nanosheets with various stoichiometric compositions are easily converted to other phases. Cobalt oxide nanomaterials can be fabricated using the as-prepared cobalt hydroxides as precursors (Huang et al. 2016; Yang et al. 2008), successfully maintaining their size and morphology. In addition, real-time phase transitions (Yoon et al. 2022; Chen et al. 2021; Jang et al. 2019) in oxidizing and reducing atmospheres (Dehghan et al 2011.; Bulavchenko et al. 2009; Potoczna-Petru et al. 2001) have also been reported, and their dynamics have been confirmed on an atomic scale. Experimental characterization using an electron microscope is a reliable and useful structural analysis technique because it provides multifaceted information on the structure, composition, and bond distance.

In this study, we investigated the phase transition induced by electron-beam irradiation in a transmission electron microscopy (TEM) column. A parallel electron beam was irradiated onto the amorphous synthesized cobalt hydroxide nanosheet to study the crystallization process. The crystal phase of the partially crystallized region was analyzed using high-resolution TEM (HR-TEM) imaging. The analysis revealed that cobalt oxides formed through the phase transition exhibited a cubic phase. Additionally, the rock salt structure, characterized by a cubic phase, was predominantly formed under the electron beam.

## Materials and methods

### Synthesis

The cobalt hydroxide nanosheets were synthesized using an aqueous nutrient solution containing 2 mM cobalt nitrate hexahydrate and 2 mM hexamethylenetetramine. Depending on the opening area of the container, a calculated amount of a chloroform solution of sodium hexadecyl sulfate was added at the water–air interface. After approximately 30 min, the container was capped and placed in a convection oven at 60 °C and 80 °C for 180 min each. The synthesized sheets at the water–air interface were scooped using a Quantifoil TEM grid for imaging.

### Characterization using SEM, AFM, and XPS

The morphology and chemical properties of the Co(OH)_2_ nanosheets were characterized using scanning electron microscopy (SEM, COXEM EN30N miniSEM), atomic force microscopy (AFM, Veeco Multimode V), and X-ray photoemission spectroscopy (XPS, Thermo Fisher K-alpha). The XPS data were obtained from the nanosheets after transferring onto a 40 nm-thick platinum coated-Si substrate to minimize the signal from the native oxide of the Si substrates.

### Transmission electron microscopy

The specimens were analysed using the transmission electron microscope (TEM, Thermo Fisher TF30ST) operated at 300 kV, at the Center for Research Facilities, Gyeongsang National University. The dose rate for the electron beam irradiation was 4 × 10^4^ e⁻/Å^2^·s.

$$\mathrm{Total}\;\mathrm{electrons}\;=\;\mathrm{Dose}\;\mathrm{rate}\;\times\;\mathrm{Area}\;\times\;\mathrm{Time}\;//\;\mathrm{Total}\;\mathrm{Electrons}\;=\;\left(4\;\times\;10^4\;\mathrm e^-/\mathrm A^2\;\mathrm s\right)\;\times\;\mathrm A\;\times\;\left(30\mathrm s\right)$$

## Results and discussion

**Fig. 1:**
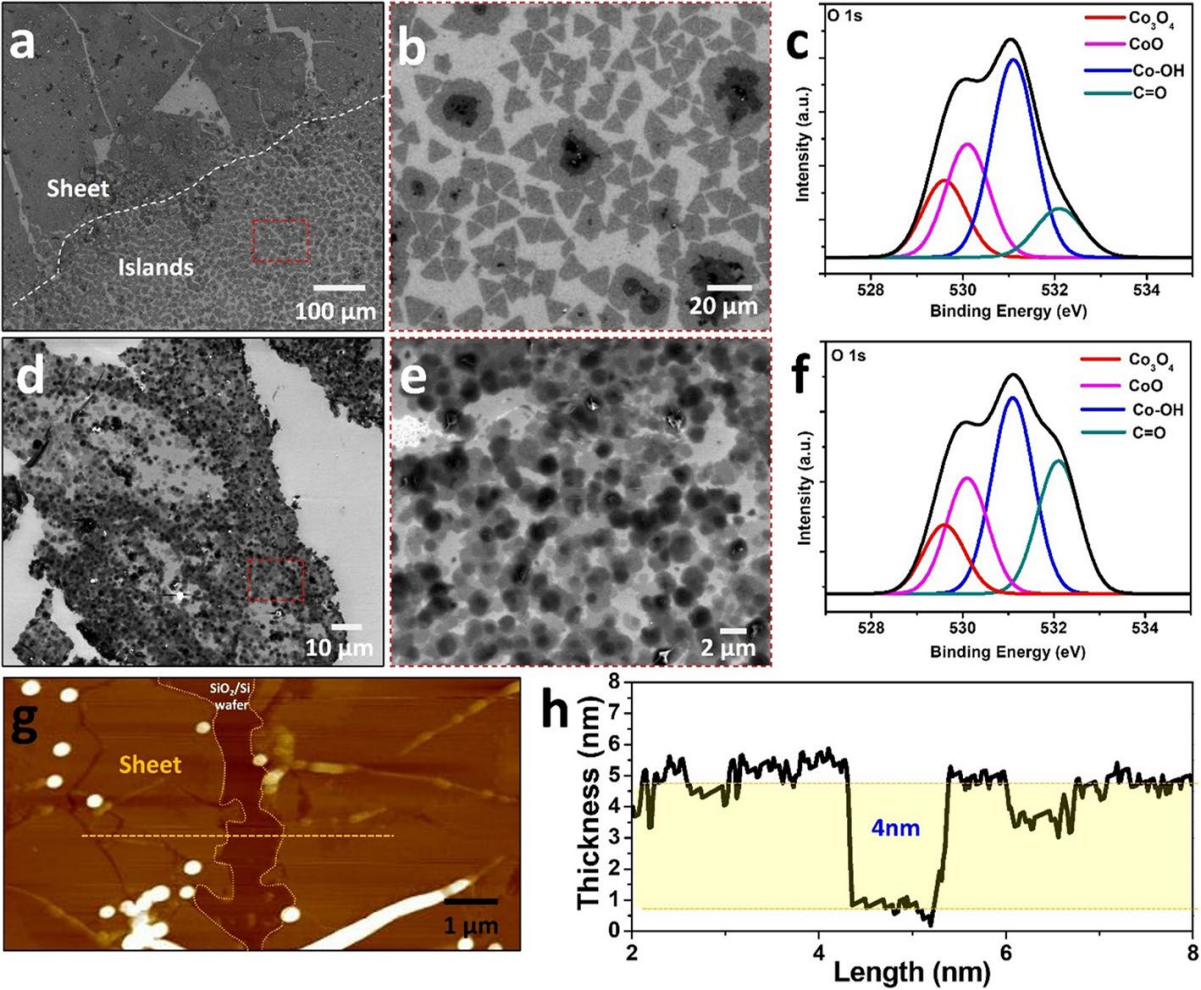
**a**, **b** SEM images and (**c**) XPS O 1 s spectra of cobalt hydroxides nanosheets synthesized at 60 °C. **d**, **e** SEM images and (**f**) XPS O 1 s spectra of cobalt hydroxides nanosheets synthesized at 80 °C. **g** Typical 2D AFM images of cobalt hydroxides nanosheets synthesized at 80 °C. **h** Line profiles taken along the yellow dashed line shown in (**g**)

Scanning electron microscopy (SEM) images show that cobalt hydroxides can be synthesized into nanosheets, as shown in Fig. 1, and that there are differences in the morphology depending on the reaction temperature (Lee et al. 2023). Based on the formation of triangular islands over a large area, the nanosheets synthesized gather to form a continuous sheet at 60 °C (Figs. 1a and b). Their chemical bonding states, which consist mainly of cobalt hydroxide and cobalt oxide, were confirmed using X-ray photoelectron spectroscopy (XPS) (Fig. 1c). Unlike the previously reported nanosheets or sheets synthesized at a reaction temperature of 80 °C, the continuous arrangement and accumulation of triangular islands remain unclear (Fig. 1d); however, approximately round sheets with contrast were continuously dominated to form a sheet (Fig. 1e). In the XPS O1s spectra (Fig. 1f), the intensity of the hydroxyl peak was the highest, and the C = O ratio appeared to be relatively high, unlike in the previous case. This is because the residue of the precursor used in synthesizing the nanosheets is higher owing to the relatively high reaction temperature.

The thickness of the synthesized cobalt hydroxide nanosheet varies depending on the reaction temperature; the higher the reaction temperature, the thicker is the synthesis [M. Lee et al. 2023]. The thickness of the nanosheets synthesized at a reaction temperature of 80 °C with high synthesis reproducibility, and a thickness of 4 nm or more was measured using atomic force microscopy (AFM) (Fig. 1g); the measured thickness was approximately 4 nm (Fig. 1h).

**Fig. 2:**
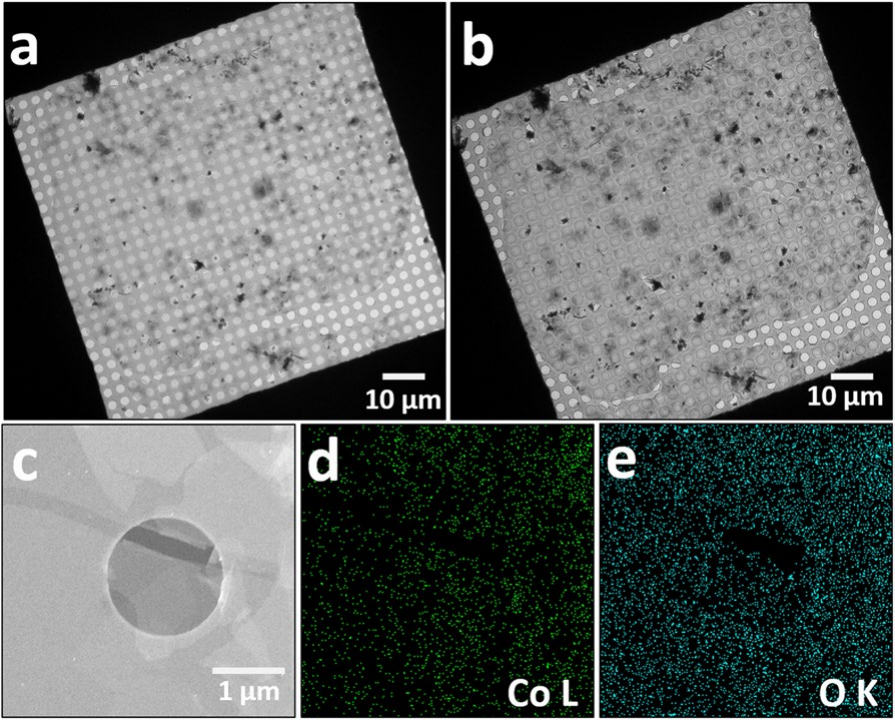
**a**, **b** In focused and overfocused low-magnification TEM images show nanosheets transferred onto a Quantifoil grid. **c** HAADF-STEM images show the sheet perfectly covered with holes in the grid. **d**, **e** EDS maps of cobalt and O elements in the same region

To analyze the crystal structure of the synthesized cobalt hydroxide nanosheets, continuous nanosheets synthesized at a reaction temperature of 80 °C were transferred to a Quantifoil grid (Fig. 2a); Fig. 2a shows an overfocused image. Although the image appears slightly distorted, it was confirmed that the transferred nanosheet covered the mesh of the grid (Fig. 2b). Figure 2c shows a high-angle annular dark-field scanning transition electron microscopy (HAADF-STEM) image of the nanosheet. Figure 2c shows the region where the nanosheets are overlapped. Element mapping was performed on the nanosheet in the same area using energy-dispersive X-ray spectroscopy (EDS), as shown in Fig. 2c, and a uniform element distribution was confirmed throughout. The EDS mapping results are provided in the table below.

**Fig. 3:**
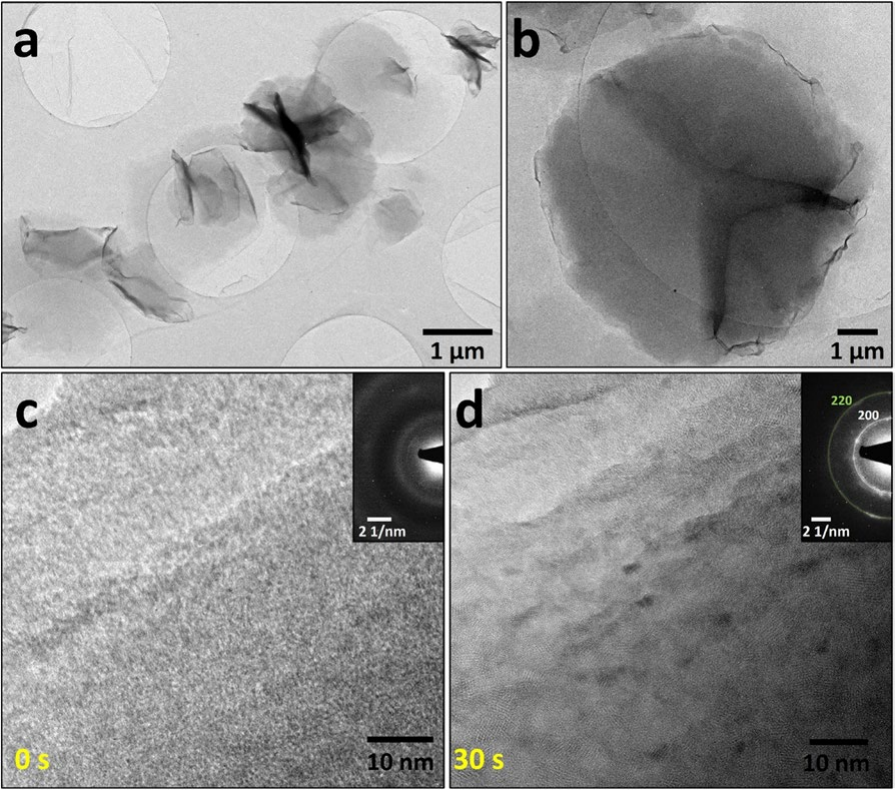
**a**, **b** TEM images show the synthesized nanosheet with some islands. **c**, **d** TEM images show the as-synthesized cobalt hydroxide nanosheet after electron-beam irradiation with each selected area diffraction patterns

The features visualized in Figs. 3a and b indicate the accumulated portions of the synthesized nanosheets, as mentioned in Figs. 1 and 2. We focused on the portions of these features with relatively thick thickness and analyzed them with high-magnification images to determine the aspect of the phase transition induced by the electron beam. HR-TEM was performed to confirm the crystallinity of the cobalt hydroxides (Fig. 3c). The pristine synthesized nanosheet was amorphous; no precision spots or ring patterns were identified in the digital diffractogram of the imaged field of view. However, after continuous irradiation with the electron beam, as shown in Fig. 3d, a partially crystallized region appeared. Additionally, a distinct spot and ring pattern were observed in the accompanying digital diffractogram. The longer the electron-beam irradiation period, the higher the crystallinity.

**Fig. 4:**
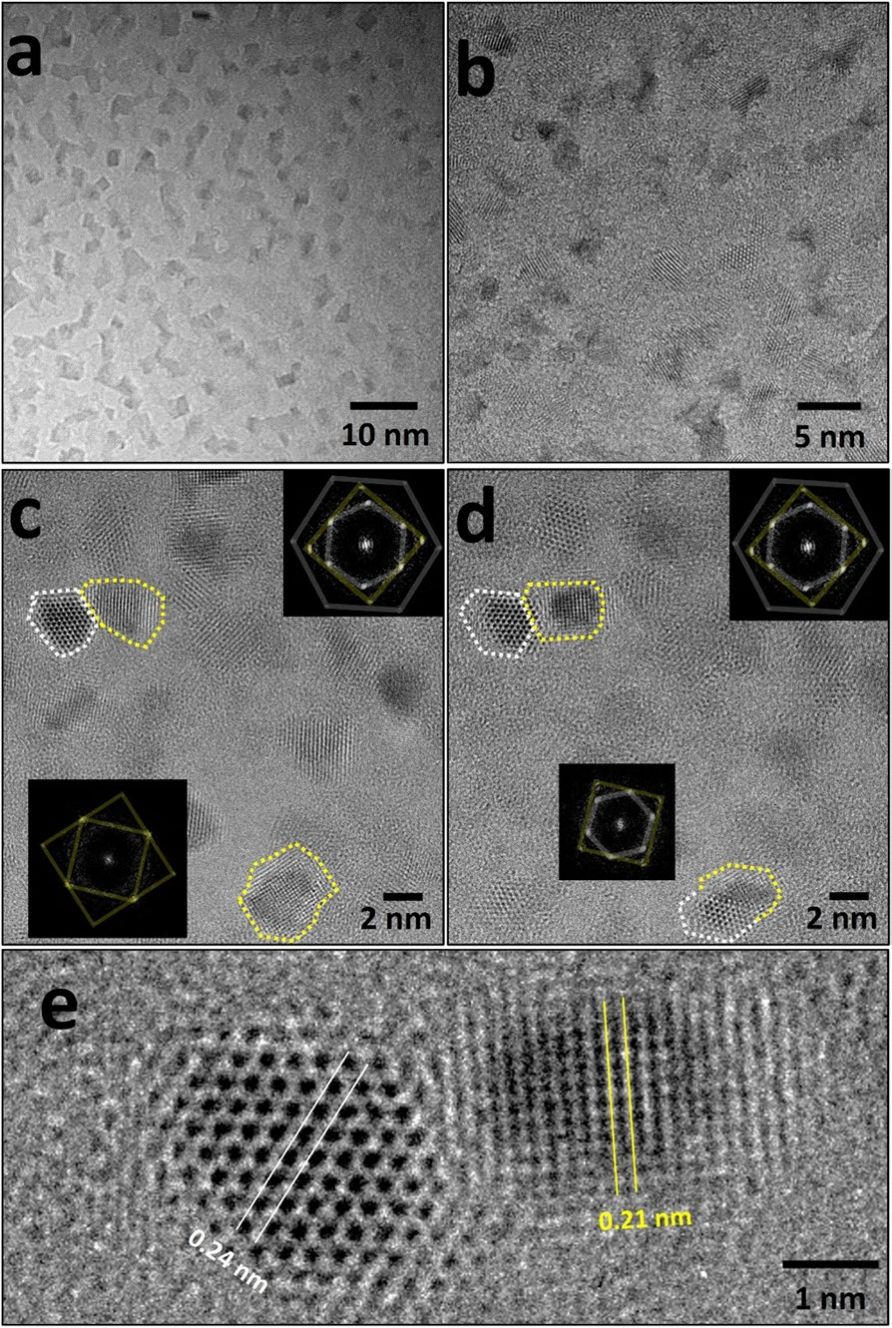
**a**, **b** TEM images show the crystallization of amorphous cobalt hydroxides by electron-beam irradiation. **c**, **d** Successive HR-TEM images show the formation of cobalt oxides in each diffractogram. **e** Magnified region of the cobalt oxides shown in (**c**) and (**d**)

As shown in Fig. 4a, crystallization proceeded with the continuous electron-beam irradiation, and this phenomenon was confirmed in the entire amorphous sheet irradiated with parallel beams (Fig. 4b). A thorough inspection of the crystallization (Figs. 4c and d) revealed distinct phases in the partial region indicated by the dashed line, and the digital diffractogram of this region suggests phase transitions from amorphous to crystalline that were caused by the electron beam. The synthesized nanosheet was composed of amorphous cobalt hydroxide, and the electron-beam irradiation caused the transformation into cobalt oxides. Cobalt hydroxide can undergo frequent phase transitions to cobalt oxides owing to external energy (Lee et al. 2023; Chen et al. 2021; Jang et al. 2019). Among them, cobalt oxides with various stoichiometric compositions are Co_3_O_4_ and CoO. Co_3_O_4_ exhibits a spinal structure, whereas CoO exhibits cubic phase [Santos et al. 2021]. The crystal structures at the top and bottom of Fig. 4c belong to CoO; the white line indicates the (111) plane, and the yellow line indicates the (200) plane. The successive images of the same area demonstrate that part of the cubic phase formed at the bottom is oriented to the (200) plane (Fig. 4d). Figure 4e shows an enlarged image of the d-spacing of 0.24 nm and 0.21 nm of the CoO phase shown at the top of Figs. 4c and d.

**Fig. 5:**
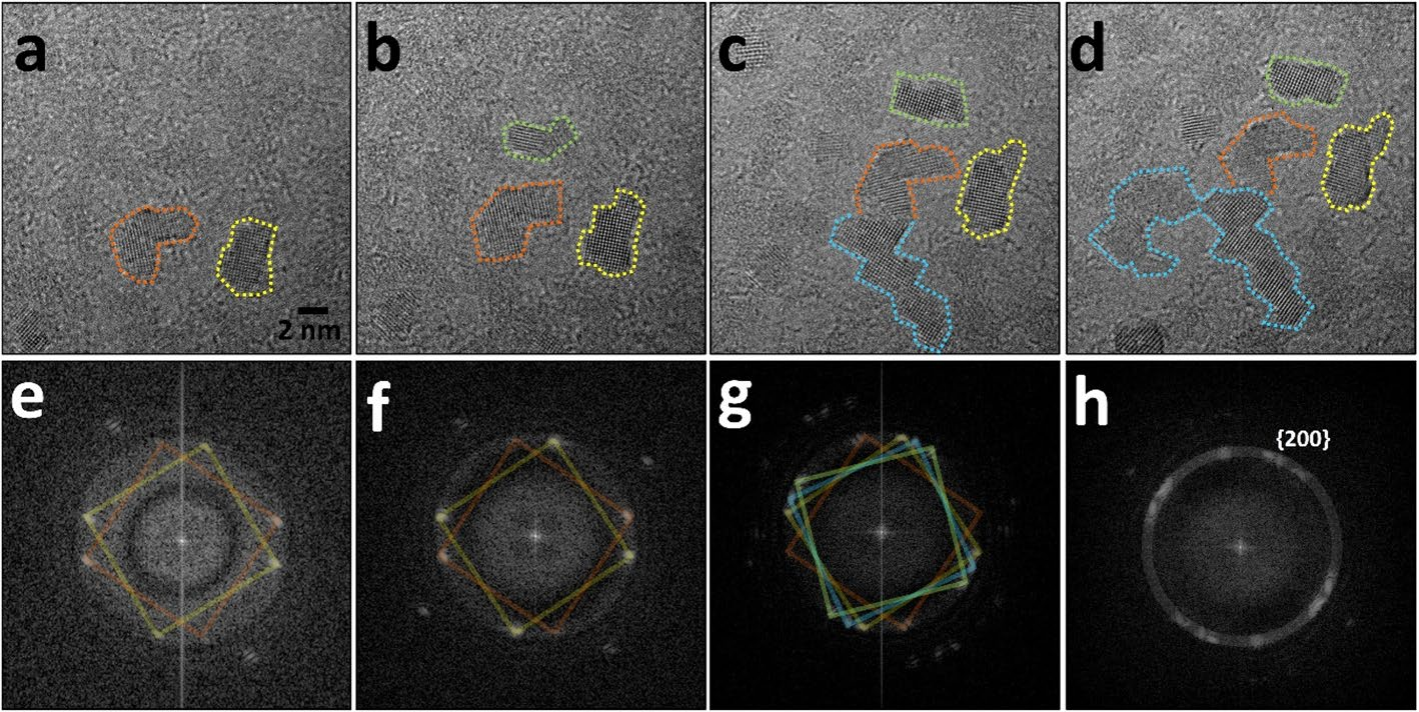
**a**–**d** Successive HR-TEM images showing the phase transition from the amorphous cobalt hydroxides to cubic cobalt oxides with (**e**)–(**h**) each digital diffractogram. Interval time between each images in sequence is 5 seconds

The partial transitions from amorphous cobalt oxide to cobalt oxide phase was confirmed using a continuous electron beam (Fig. 5). The transition phase consisted of cubic phase, which was being dominant. The areas marked with the colored dashed lines indicate the several regions of the cubic cobalt oxides. Because the transitioned cubic phase is nanosized, the digital diffractogram shows an increase in the number of spots owing to the different orientations of those continuously transferred in the random area.

## Conclusions

We investigated the crystallization behavior and phase transitions formed by continuous electron-beam irradiation on cobalt hydroxide nanosheets. The synthesized amorphous cobalt hydroxide nanosheets with a thickness of approximately 4 nm were crystallized using parallel electron-beam irradiation. Partially crystallized cobalt monoxide (cubic phase) resulted from crystallization by continuous electron-beam irradiation, which expanded the crystallized region and accelerated the transition to cubic cobalt oxide. These behaviors were confirmed through HR-TEM imaging, and the results demonstrate that the phase transition of cobalt oxides with various stoichiometric compositions can be realized and accelerated by electron beams.

## Data Availability

The datasets used and/or analyzed during the study are available from the corresponding author upon reasonable request.

## Abbreviations

XPS: X-ray Photoelectron Spectroscopy
SEM: Scanning Electron Microscopy
AFM: Atomic Force Microscopy
HAADF-STEM: High Angle Annular Dark Field Scanning Transition Electron Microscopy
EDS: Energy Dispersive X-ray Spectroscopy
HR-TEM: High-resolution Transition Electron Microscopy
